# Black Truffles Affect *Quercus aliena* Physiology and Root-Associated *nirK*- and *nirS*-Type Denitrifying Bacterial Communities in the Initial Stage of Inoculation

**DOI:** 10.3389/fmicb.2022.792568

**Published:** 2022-04-28

**Authors:** Zongjing Kang, Xiaolin Li, Yan Li, Lei Ye, Bo Zhang, Xiaoping Zhang, Petri Penttinen, Yunfu Gu

**Affiliations:** ^1^Department of Microbiology, College of Resources, Sichuan Agricultural University, Chengdu, China; ^2^Soil and Fertilizer Institute, Sichuan Academy of Agricultural Sciences, Chengdu, China; ^3^Ecosystems and Environment Research Programme, University of Helsinki, Helsinki, Finland

**Keywords:** *Tuber indicum*, *Tuber melanosporum*, *Quercus aliena*, ectomycorrhizosphere, denitrifying bacteria, growth of host plants, soil properties

## Abstract

Truffles (*Tuber* spp.) are edible ectomycorrhizal fungi with high economic value. Bacteria in ectomycorrhizosphere soils are considered to be associated with the nutrient uptake of truffles and hosts. Whether *Tuber* spp. inoculation can affect the growth of *Quercus aliena*, the ectomycorrhizosphere soil, and the rhizosphere *nirK* and *nirS*-denitrifier communities at the ectomycorrhizae formation stage is still unclear. Therefore, we inoculated *Q. aliena* with the black truffles *Tuber melanosporum* and *Tuber indicum*, determined the physiological activity and morphological indices of *Q. aliena* seedlings, analyzed the physicochemical properties of ectomycorrhizosphere soils, and applied DNA sequencing to assess the *nirK* and *nirS*- denitrifier community structure in ectomycorrhizosphere soils. Peroxidase activity was higher in the seedlings inoculated with *T. melanosporum* than in the *T. indicum* inoculation and uninoculated control treatments. The available phosphorus contents were lower and nitrate contents were higher in those with truffle inoculation, and *T. melanosporum* treatment differed more from the control than the *T. indicum* treatment. The richness of the *nirK*-community was highest in the *T. indicum* treatment and lowest in the uninoculated treatment. The differences in *nirK*-community composition across treatments were not statistically significant, but the *nirS* communities were different. The *nirS*-type bacteria correlated with three environmental factors (pH, available phosphorus, and nitrate contents), whereas the *nirK*-type bacteria were only associated with the nitrate contents. Generally, this work revealed that inoculation with *Tuber* spp. would change a few nutrient contents and richness of *nirK*-type bacteria and had little effects on growth of *Q. aliena* seedlings in the initial stage of inoculation. The results of this study may provide in-depth insights into the relationships between *Tuber* spp. and hosts, which should be taken into account when developing truffle production methods.

## Introduction

*Tuber* spp., commonly known as truffles, belong to the family *Tuberaceae* in the order Pezizales, phylum Ascomycota. The truffle fruiting bodies are valuable because of their specific aroma and are expensive delicacies due to the decreasing yields of wild truffles and unpredictability of cultivation (Allen and Bennett, 2021). *Tuber* spp. coexist and interact with their host plants through the formation of ectomycorrhizae. The major host plants of *Tuber* spp. include various *Corylaceae* species, among them, *Pinus* and *Quercus* (Geng et al., 2009; Garcia-Barreda et al., 2015; Wan et al., 2016). The growth and development of truffles and their hosts are closely associated as the truffles form a mass of mycelia in the soil surrounding the host plant roots (Suz et al., 2010a). Ectomycorrhizal fungi may improve the growth of their hosts in oligotrophic environments and under drought (Alvarez et al., 2010), such as increasing average plant height, ground diameter growth, and disease resistance (Liese et al., 2017). *Quercus aliena* Blume is an oak specie native to East Asia, including southwest China, which is a truffle producing region (Li et al., 2014). *Q. aliena* with high adaptability to dynamic environment is a suitable host species for *Tuber* spp. (Li et al., 2018). However, there is little information on how *Tuber* species affect the growth of *Q. aliena*.

Bacteria in ectomycorrhizosphere soils were considered to be associated with the mycelium colonization and the formation of volatile aroma compounds of truffle (Splivallo et al., 2015). The formation of ectomycorrhizae is accompanied by changes in soil microbial communities and soil properties (García-Montero et al., 2006; Barbieri et al., 2007). For example, inoculation with *Tuber* spp. changed the nitrogen content in ectomycorrhizosphere (Li et al., 2018; Yang et al., 2019), possibly due to the enrichment of nitrogen cycle-related bacteria in the truffle ascocarps (Barbieri et al., 2010; Vahdatzadeh et al., 2015; Monaco et al., 2021), ectomycorrhizae, and mycorrhizosphere (Beatrice et al., 2012; Le-Roux et al., 2016). Denitrification is a key nitrogen-transforming process which results in nitrogen loss in soil (Bárta et al., 2017). The taxonomic composition of denitrifying bacteria, denitrifiers correlated with the content of ammonia-nitrogen (NH_4_^+^-N) and nitrate-nitrogen (NO_3_^–^-N) that can be directly absorbed by *Tuber* mycelia (Montanini et al., 2002) and its hosts (Marcel et al., 2018). *Pseudomonas*, *Bradyrhizobium*, and *Ensifer* were the predominant bacterial genera associated with truffle (Barbieri et al., 2007; Benucci and Bonito, 2016; Deveau et al., 2016). And these genera include many denitrifiers such as *Pseudomonas aeruginosa* (Zhang et al., 2020), *Ensifer meliloti* (Chan and Mccormick, 2004; Torres et al., 2014, 2018), *Bradyrhizobium japonicum* (Mesa et al., 2010), *Pseudomonas stutzeri* (Wittorf et al., 2018), and *Bradyrhizobium oligotrophicum* (Cristina and Kiwamu, 2011). These carry either of the nitrite reductase genes *nirK* and *nirS*, which previous studies have indicated to be widely distributed in *Tuber* spp. ectomycorrhizosphere soils.

The community structure of *nirK*- and *nirS*- denitrifiers is affected by environmental factors, including availability of carbon sources and nitrate concentration (Azziz et al., 2017). However, whether the specific ectomycorrhizosphere soil properties (García-Montero et al., 2006; Barbieri et al., 2007; Li et al., 2018) reregulate the structure of *nirK*- and *nirS*-carrying bacterial communities is still unclear. In addition, the effects of the *Tuber* spp. inoculation on the primary nutrient in ectomycorrhizosphere soil and growth of hosts during ectomycorrhizae formation stage remain to be assessed. Therefore, we inoculated *Q. aliena* with the black truffles *Tuber melanosporum* Vittad. and *Tuber indicum* Vittad., determined the physiological activity and morphological indices of *Q. aliena* seedlings, analyzed the physicochemical properties of ectomycorrhizosphere soils, and applied DNA sequencing to assess the *nirK* and *nirS*- denitrifier community structure in ectomycorrhizosphere soils. Our aims were to define the effect of *Tuber* spp. inoculation on the growth of *Q. aliena* seedlings in the initial stage of ectomycorrhizae formation, and to elucidate the environmental factors affecting the denitrifier community structure in ectomycorrhizosphere soils.

## Materials and Methods

### Cultivation of *Quercus aliena* Seedlings

*Tuber melanosporum* were from France and *T. indicum* were from Huidong, China. *Q. aliena* seeds were obtained from Yunnan Academy of Forestry Sciences, China. Seeds were soaked in fresh water for approximately 20 h, sterilized in 0.3% potassium permanganate for 30 min, and rinsed with distilled water until the rinse water became colorless. River sand was sterilized for 90 min at 121^°^C and the seeds were germinated in damp sand for 1 month. The germinated seeds were sown in pots filled with a sterilized mixture of vermiculite, perlite, and water at a ratio of 1:1:1 (v/v/v). The pots were placed in a plastic greenhouse with a daytime temperature of 23–25°C and a night-time temperature of 16–20°C and watered with sterile water to maintain soil moisture at 25%. After 1 month, the *Q*. *aliena* seedlings were transplanted into nursery pots full with sterilized mixture. The truffles inoculum and inoculation were done as described previously (Kang et al., 2020). The seedlings were inoculated with *T. melanosporum* (mel.ali) or *T. indicum* (ind.ali), and uninoculated *Q. aliena* seedlings served as a control treatment (CK.ali). Sixty seedlings per treatment were grown in a greenhouse for 180 days. The moisture content in the substrate was maintained at about 50% by watering with tap water every 2–3 days. In the greenhouse, the average temperature was 22.2°C (10.7–35.6°C) and the average humidity was 78.8% (45–100%) from March to July, and 24.0°C (12.7–38.7°C) and 82.07% (39–100%) from July to November.

### Morphological Identification and Morpho-Anatomy of Ectomycorrhizae and Sample Collection

Six seedlings per treatment were randomly sampled 6 months after inoculation. Three seedlings were used for ectomycorrhizal identification, and the other three were used to analyze the development of seedlings. Soil was removed from the rootlets by gentle shaking, and the bulk soil was collected to determine soil properties. The soil still adhering to the roots (<3 mm thick soil) were scraped with sterile tweezers and collected as the rhizosphere soil. Approximately 3.0 g of rhizosphere soil per seedling was stored at -80°C for microbial analysis. The roots of seedlings were rinsed with water and observed under microscope. The morpho-anatomy of the ectomycorrhizae were characterized by fixing with paraffin, followed by transverse and longitudinal slicing into 3 μm thick sections using a revolving slicer (Leica, RM2016, Wetzlar, Germany). The sections were stained with fast green for 3 min and safranin O for 0.5–2 min. The images of cross and longitudinal sections were captured using a Pannoramic 250 fast length adjustment of short reads (FLASH) digital section scanner (3DHIESTECH, Budapest, Hungary).

### Determining Physiological Indices and Soil Properties

To measure the growth of the *Q. aliena* seedlings, the shoot height, stem diameter, root weight, crown weight, root dry weight, and crown dry weight of the seedlings were determined (Supplementary Table 1). Seedling index and root crown ratio (Moore et al., 2002) were calculated using Equations 1 and 2:


$$S\,e\,e\,d\,l\,i\,n\,g\,i\,n\,d\,e\,x=\left(\frac{s\,t\,e\,m\,d\,i\,a\,m\,e\,t\,e\,r}{s\,h\,o\,o\,t\,h\,e\,i\,g\,h\,t}+\frac{r\,o\,o\,t\,w\,e\,i\,g\,h\,t}{c\,r\,o\,w\,n\,w\,e\,i\,g\,h\,t}\right)$$

(1) ×t⁢o⁢t⁢a⁢l⁢w⁢e⁢i⁢g⁢h⁢t

(2) $$R\,o\,o\,t\,c\,r\,o\,w\,n\,r\,a\,t\,i\,o=\frac{r\,o\,o\,t\,d\,r\,y\,w\,e\,i\,g\,h\,t}{c\,r\,o\,w\,n\,d\,r\,y\,w\,e\,i\,g\,h\,t}$$

Young roots (0–5mm in diameter and 0–10cm from apical) from three seedlings per treatment were sampled for measuring physiological indices. Root activity was measured using triphenyltetrazolium chloride (TTC) staining (Zhang et al., 2013), superoxide dismutase (SOD) activity was measured using the NBT method (Fridovich, 2011), and peroxidase (POD) activity was determined using the guaiacol colorimetric method (Meloni et al., 2003).

Total nitrogen (TN), nitrate-nitrogen (NO_3_^–^-N), ammonium-nitrogen (NH_4_^+^-N), available phosphorus (AP), available potassium (AK), and organic matter (OM) contents, and pH of the soil were measured as described previously (Hill et al., 2003).

### DNA Extraction and Sequencing

Deoxyribonucleic acid was extracted from three replicate 1.0 g rhizosphere soil samples per treatment using the FastDNA Spin Kit (MP Biomedicals, Santa Ana, CA, United States). The quality of extracted DNA was analyzed using gel electrophoresis. The DNA extracts were stored at −20°C.

The *nirS* and *nirK* fragments were amplified using primers *nirK*F (5′-TCATGGTGCTGCCGCGY- GANGG-3′), *nirK*R (5′-GAACTTGCCGGTKGCCCAGAC-3′) (Yan et al., 2003), *nirS*F (5′-TT- CRTCAAGACSCAYCCGAA-3′), and *nirS*R (5′-CGTTGAACTTRCCGG-3′) (Hill et al., 2003). The 20 μl PCR reactions contained 0.5 μl primer, 10 μl SYBR Green PCR master mix (Power SYBR*^R^* Green PCR master mix, Applied Biosystems, United States), and 2 μl of 5 ng μl^–1^ template DNA. The PCR procedure included an initial denaturation at 94°C for 4 min, 30 cycles of 15 s at 94°C, 15 s at 55°C, and 30 s at 72°C, and a final extension at 72°C for 10 min (Bárta et al., 2010). Amplified fragments in 2 μl of the PCR reaction were separated by electrophoresis in a 1.5% agarose gels that were extracted using an Axygen DNA Gel Extraction Kit (Axygen Inc., Corning, NY, United States), and quantified using a Quant-iT PicoGreen dsDNA Assay Kit and a FLx800 microplate reader (BioTek, Vermont, United States). Sequencing libraries were prepared using a TruSeq Nano DNA LT Library Prep Kit and quantified using a Promega QuantiFluor fluorescence quantification system (Promega Corporation, Madison, WI, United States). The libraries were sequenced using an Illumina MiSeq sequencer (Personalbio, Shanghai, China) and 2 × 300 bp paired-end reads were generated.

### Sequence Analysis

Paired-end reads were merged using FLASH (Mago and Salzberg, 2011) and quality filtered using Quantitative Insights Into Microbial Ecology (QIIME) and Trimmomatic (Caporaso et al., 2010). The sequences were assigned to operational taxonomic units (OTUs) at 97% similarity level using Uparse software. The 30,800-73,109 *nirK* sequence reads per sample were classified into 1,734-3,125 OTUs per sample, and the 56,427-81,655 *nirS* gene sequence reads into 585-1145 OTUs (Supplementary Figure 1). Rarefaction curves were generated using QIIME (Supplementary Figure 2). The alpha diversity was evaluated by calculating five indices using QIIME, including Chao1, Shannon, Simpson ACE, and Observed species. The OTUs were assigned to taxa using the Ribosomal Database Project Classifier^1^ (Wang et al., 2007) based on the functional gene pipeline & repository database (Release7.3).^2^ The taxonomic composition was visualized using R v 3.5.0.^3^ The sequence data were submitted to the NCBI Sequence Read Archive (SRA) database with the accession numbers SRX6578870–SRX6578878.

### Statistical Analysis

The normality of data were tested using the Shapiro-Wilk test (Supplementary Figure 3) and one-way ANOVA with Duncan’s multiple range test. Least significant difference (LSD) tests were done using Statistical Package for the Social Sciences (SPSS) version 10.0 for Windows (IBM SPSS Inc., Chicago, IL, United States). Differences were considered statistically significant at *p* < 0.05. After removal of singleton and unidentified OTUs, the multivariate homogeneity of dispersions was tested with 999 permutations in the R package vegan v2.5-7 in R 4.1.2 (Oksanen et al., 2020). Differences in beta-diversity were tested using permutational multivariate analysis of variance (PERMANOVA) and pairwise PERMANOVA in the R packages vegan and pairwise Adonis (Anderson, 2006) (Supplementary Figure 4). Weighted UniFrac based beta-diversity was visualized using non-metric multidimensional scaling (NMDS). We standardized soil properties and *nirK*- and *nirS*-bacterial genera using z-score, and the soil properties associated with the variation in *nirK* and *nirS* communities were analyzed using redundancy analysis (RDA) in Canoco V 4.5 (Center for Biometry, Wageningen, Netherlands).

## Results

### The Characteristics of Ectomycorrhizae, Seedlings, and Soil

**FIGURE 1:**
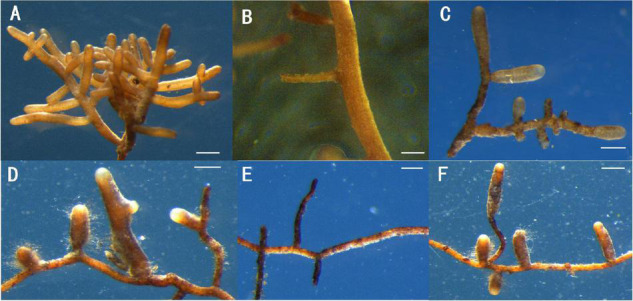
Two ectomycorrhizae and roots of *Quercus aliena* without *Tuber* species partner, *Scale bar* = 1 mm. **(A,D)** Ectomycorrhizae of *Q. aliena* seedlings with *Tuber indicum*; **(B,E)** root of *Q. aliena* without *Tuber* partner; **(C,F)** Ectomycorrhizae of *Q. aliena* seedling with *Tuber melanosporum*.

**FIGURE 2:**
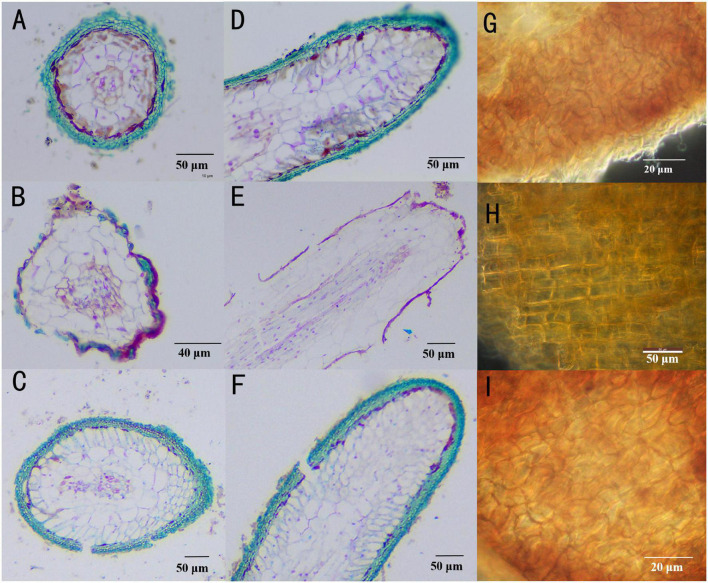
Structural characteristics of *Quercus aliena* root tips with or without *Tuber* partner. **(A)** Cross section of *Tuber indicum* ectomycorrhizae, *Scale bar* = 50 μm; **(B)** *Q. aliena* roots without *Tuber* partner, *Scale bar* = 40 μm; **(C)** *Tuber melanosporum* ectomycorrhizae, *Scale bar* = 50 μm; **(D)** longitudinal section of *T. indicum* ectomycorrhizae *Scale bar* = 50 μm; **(E)** *Q. aliena* roots without *Tuber* partner, *Scale bar* = 50 μm; **(F)** *T. melanosporum* ectomycorrhizae, *Scale bar* = 50 μm; **(G)** mantle cell of *T. indicum* ectomycorrhizae, *Scale bar* = 20 μm; **(H)** cell of root tips without *Tuber* partner, *Scale bar* = 50 μm. **(I)** mantle cell of *T. melanosporum* ectomycorrhizae, *Scale bar* = 20 μm.

Inoculation with *T. melanosporum* and *T. indicum* resulted in typical ectomycorrhizae on the *Q. aliena* seedlings (Figure 1). Two ectomycorrhizae were monopodial or binary branched and were yellowish-brown, and some ectomycorrhizae formed abundant transparent mycelium. The outer green cells in images of cross (Figures 2A,C) and longitudinal (Figures 2D,F) were the outer mantle cells, the root without *Tuber spp*. inoculation had no mantle cell (Figures 2B,E). Closer examination revealed uneven puzzle-like patterns on the ectomycorrhizal tips (Figures 2G,I), which were different from rectangle cell in root tips (Figure 2H). The average seedling index ranged from 7.96 to 9.48, and the root crown ratio ranged from 2.58 to 3.46 (Table 1). The SOD activity and root activity ranged from 3.23 to 4.12 U(gh)^–1^ FW and 21.20 to 25.13 μg TTF(gh)^–1^. The seedling index, root crown ratio, SOD activity, and root activity were on the same level in three treatments. The POD activity was higher in the *T. melanosporum* inoculated seedlings than in the not inoculated and *T. indicum* inoculated seedlings (*p* < 0.05). However, the ind.ali did not differ significantly in POD activity from CK.ali.

**TABLE 1:** Physiological indices of *Quercus aliena* seedlings.

Sample	Seedling index	Root crown ratio	Root activity μg TTF/(g⋅h)	SOD activity U/(g⋅h) FW	POD activity U/(g⋅min) FW
CK.ali	9.48 ± 3.73a	3.46 ± 0.76a	25.13 ± 10.78a	3.23 ± 0.77a	3.78 ± 0.23b
mel.ali	7.96 ± 1.12a	2.79 ± 0.50a	21.20 ± 0.55a	3.69 ± 0.47a	7.97 ± 0.16a
ind.ali	9.12 ± 2.02a	2.58 ± 0.49a	22.51 ± 12.31a	4.12 ± 1.23a	3.20 ± 1.36b
*P-value*	0.7549	0.2514	0.8771	0.5025	0.0007
*F-value*	0.2948	1.7532	0.134	0.7734	31.413

*Values are mean ± standard deviation (n = 3). Values followed by different lowercase letters indicate significant differences (p < 0.05) among samples within a line. CK.ali, rhizosphere soil of Q. aliena without Tuber partner; mel.ali, rhizosphere soil of Q. aliena with T. melanosporum; ind.ali, rhizosphere soil of Q. aliena with T. indicum.*

The average pH, content of OM, NH_4_^+^-N content, and the AK content in the control treatment were not significantly different from *T. melanosporum* and *T. indicum* treatments (Table 2). The NO_3_^–^-N content ranged from 5.04 to 8.71 g kg^–1^, AP content ranged from 9.88 to 19.95 mg kg^–1^, and TN content from 0.82 to 0.90 g kg^–1^. The content of NO_3_^–^-N and AP in *Tuber* spp. treatments were higher in the uninoculated treatment. Furthermore, these significantly higher in *T. melanosporum* treatment than in the *T. indicum* treatment (*p* < 0.05). The total nitrogen content was significantly higher in the *T. melanosporum* treatment than in the *T. indicum* treatment (*p* < 0.05), but there was no significant difference between *Tuber* spp. treatments and control treatment.

**TABLE 2:** Physical and chemical properties of soil samples.

Sample	pH	OM (g/kg)	TN (g/kg)	NH_4_^+^-N (mg/kg)	NO_3_^–^-N (mg/kg)	AK (mg/kg)	AP (mg/kg)
CK.ali	9.56 ± 0.03a	36.12 ± 1.88ab	0.89 ± 0.02ab	10.58 ± 0.45a	5.04 ± 0.07c	155.91 ± 2.8ab	19.95 ± 2.26a
mel.ali	9.53 ± 0.04a	34.34 ± 2.74ab	0.90 ± 0.07a	10.77 ± 0.23a	8.71 ± 0.14a	168.83 ± 12.82a	9.88 ± 2.26c
ind.ali	9.47 ± 0.05a	36.95 ± 1.51a	0.82 ± 0.01b	10.55 ± 0.53a	6.42 ± 0.09b	170.45 ± 39.46a	15.26 ± 4.74b
*P-value*	0.8034	0.3638	0.1068	0.8057	<0.0001	0.7310	0.0036
*F-value*	3.8000	1.2025	3.3225	0.2240	1040.6745	0.3303	16.5763

*Values are mean ± standard deviation (n = 3). Values followed by different lowercase letters indicate significant differences (p < 0.05) among samples within a line.*

### The *nirK*- and *nirS*-Communities

In the rhizospheres, the diversity of the *nirK*-community was higher in the *T. indicum* treatment than in the uninoculated treatment (*p* < 0.05) (Table 3). The richness of the *nirK*-community was highest in the *T. indicum* treatment and lowest in the uninoculated treatment (*p* < 0.05). The diversity and richness of the *nirS*-communities were on the same level in all the treatments.

**TABLE 3:** Community richness and diversity indices of *nirK-* and *nirS*-type denitrifying bacteria in the rhizosphere soil of *Quercus aliena* with or without *Tuber* partner.

Sample		Chao1	ACE	Simpson	Shannon	Observed species
*nirK*	CK.ali	1169.71 ± 97.04c	1181.71 ± 110.15c	0.96 ± 0.02a	7.04 ± 0.44bc	1735.33 ± 136.03c
	mel.ali	1561.48 ± 154.67b	1581.22 ± 169.07b	0.97 ± 0.02a	7.82 ± 0.49ab	2306.67 ± 158.65b
	ind.ali	2025.68 ± 128.09a	2136.11 ± 115.51a	0.98 ± 0.02a	8.21 ± 0.41a	3126.33 ± 106.92a
*nirS*	CK.ali	1098.66 ± 222.75c	1110.22 ± 224.09c	0.98 ± 0.01a	7.38 ± 0.46abc	1373.33 ± 161.57d
	mel.ali	928.09 ± 138.54c	933.41 ± 130.9c	0.98 ± 0a	7.31 ± 0.48bc	1290.33 ± 245.3d
	ind.ali	928.01 ± 318.05c	940.69 ± 329.72c	0.96 ± 0.01a	6.61 ± 0.47c	1204.33 ± 321.43d
Levene statistic	*P-value*	<0.0001	<0.0001	0.1079	0.0145	<0.0001
	*F-value*	15.2034	17.0592	2.3195	4.5700	41.3017

*Values are mean ± standard deviation (n = 3). Values followed by different lowercase letters indicate significant differences (p < 0.05) among samples within a line. ACE, abundance-based coverage estimator; Chao1, Chao1 richness estimator; Shannon, Shannon-Weiner index; Simpson, Simpson index.*

**FIGURE 3:**
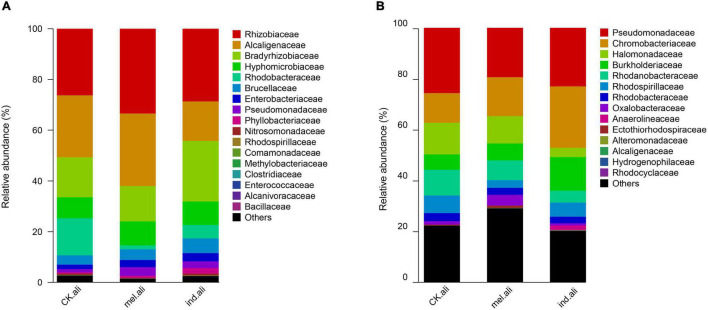
Family level taxonomic composition of **(A)** *nirK*- and **(B)** *nirS*-type denitrifier communities in the rhizosphere soil of *Quercus aliena*. CK.ali, uninoculated *Q. aliena*; ind.ali, *Q. aliena* inoculated with *Tuber indicum*; and mel.ali, *Q. aliena* inoculated with *Tuber melanosporum*.

**FIGURE 4:**
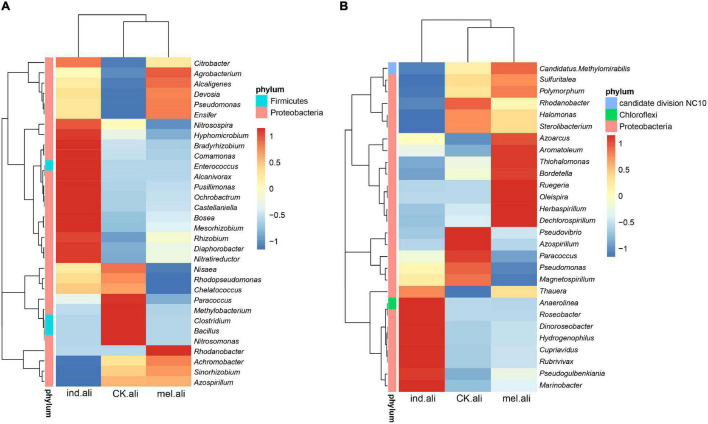
Heat-maps of the most abundant **(A)** *nirK*- and **(B)** *nirS*-type denitrifying bacterial genera in rhizosphere soil of *Quercus aliena*. CK.ali, uninoculated *Q. aliena*; ind.ali, *Q. aliena* inoculated with *Tuber indicum*; and mel.ali, *Q. aliena* inoculated with *Tuber melanosporum*.

The identified *nirK* were mostly assigned to phyla Proteobacteria (98.1–99.9%) and Firmicutes (<0.1%), and to families *Rhizobiaceae*, *Bradyrhizobiaceae*, *Alcaligenaceae*, *Hyphomicrobiaceae*, and *Rhodobacteraceae* (Figure 3A). The *Achromobacter*, *Bosea*, *Sinorhizobium*, *Rhizobium*, *Devosia*, *Ochrobactrum*, *Paracoccus*, and *Citrobacter* were dominant genera in three treatments (Supplementary Table 2). *Achromobacter* (13%) was a predominant denitrifer in *T. melanosporum* treatment, while *Nitratireductor*, *Diaphorobacter*, *Mesorhizobium*, *Bosea*, and *Ochrobactrum* were predominant denitrifers in *T. indicum* treatment (Figure 4A). The identified *nirS* were mostly assigned to phyla Proteobacteria (97.5–82.5%), Chloroflexi (0–5.2%), and Candidate division NC10 (0–.3%), and to families *Pseudomonadaceae*, *Chromobacteriaceae*, *Halomonadaceae*, *Burkholderiaceae*, and *Rhodanobacteraceae* (Figure 3B). *Pseudomonas*, *Pseudogulbenkiania*, *Halomonas*, *Cupriavidus*, *Magnetospirillum*, *Rhodanobacter*, *Rubrivivax*, and *Azoarcus* were dominant *nirS*-type bacterial genera (Supplementary Table 2). *Dechlorospirillum*, *Herbaspirillum*, *Oleispira*, and *Ruegeria* were predominant denitrifers in *T. melanosporum* treatment, while *Marinobacter*, *Rubrivivax*, *Cupriavidus*, *Hydrogenophilus*, and *Dinoroseobacter* were predominant denitrifers in *T. indicum* treatment (Figure 4B).

**FIGURE 5:**
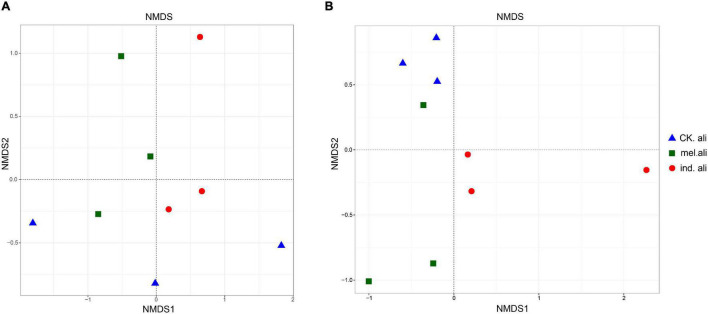
Non-metric multidimensional scaling (NMDS) plots of **(A)** *nirK*- and **(B)** *nirS*-type denitrifying bacterial communities in rhizosphere soil of *Quercus aliena*. CK.ali, rhizosphere soil of *Q. aliena* without *Tuber* partner; mel.ali, ectomycorrhizosphere soil of *Q. aliena* with *Tuber melanosporum* partner; and ind.ali, ectomycorrhizosphere soil of *Q. aliena* with *Tuber indicum* partner.

**FIGURE 6:**
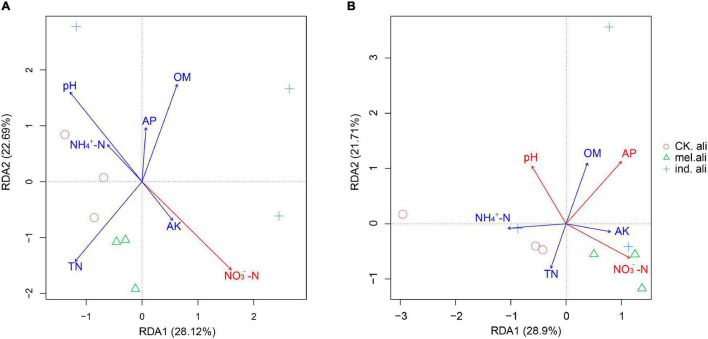
Redundancy analysis (RDA) between **(A)** *nirK*- and **(B)** *nirS*-type denitrifying bacterial communities and soil factors in the rhizosphere soil of *Quercus aliena*. CK.ali, rhizosphere soil of *Q. aliena* without *Tuber* partner; mel.ali, ectomycorrhizosphere soil of *Q. aliena* with *Tuber melanosporum* partner; and ind.ali, ectomycorrhizosphere soil of *Q. aliena* with *Tuber indicum* partner.

In the weighted UniFrac-based NMDS, the *nirK* communities were not clearly separated into treatment-specific groups. The samples in *T. indicum* and *T. melanosporum* treatments were similar but different from those in the control treatment (Figure 5A). The *nirS* communities showed that three samples of the control treatment were collected in a group, and only two samples in *T. indicum* and *T. melanosporum* treatment were similar. The control treatment was different with *Tuber* spp. treatments, and *T. indicum* treatment was also different with *T. melanosporum* treatment (Figure 5B). In the redundancy analysis, the axis RDA1 accounted for 28.12% and RDA2 for 22.69% of the variation in *nirK*-community composition (Figure 6A). The differences in *nirK*-community composition were associated with differences in NO_3_^–^-N content (*r*^2^ = 0.6646, *p* = 0.026). RDA1 accounted for 28.90% and RDA2 for 21.71% of the variation in *nirS*-community composition (Figure 6B). The differences in *nirS*-community composition were associated with differences in pH (*r*^2^ = 0.5875, *p* = 0.043) and available phosphorus (*r*^2^ = 0.9228, *p <* 0.001) and NO_3_^–^-N (*r*^2^ = 0.6778, *p* = 0.017) content.

## Discussion

We assessed how *T. melanosporum* and *T. indicum* inoculations affect the growth of *Q. aliena* seedlings, the properties of rhizosphere soil, and the associated *nirK*- and *nirS*-denitrifier communities in the initial stage of ectomycorrhizae formation. The morphology of *T. melanosporum* and *T. indicum* ectomycorrhizae were similar to the ectomycorrhizae of other *Tuber* spp. and inoculated *Quercus* spp. (Perez et al., 2007; Suz et al., 2010b; Li et al., 2018). Likewise, the cross and longitudinal sections of the ectomycorrhizae were similar to those of the other *Tuber* spp.–*Quercus* spp. ectomycorrhizae (Benucci et al., 2012; Deng et al., 2014; Zhang et al., 2019), and the uneven puzzle-like patterns in the root tips were similar to those of *T. melanosporum*– and *T. indicum*–*Quercus* spp. ectomycorrhizae (Deng et al., 2014; Huang et al., 2020).

Similar to inoculating *Pinus armandii* and *Carya illinoinensis* with *Tuber* spp. (Zhang et al., 2020; Huang et al., 2021), inoculating *Q. aliena* with *T. melanosporum* and *T. indicum* barely affected the plant parameters 6 months after inoculation. However, earlier studies showed that inoculation with *Tuber* spp. improved the growth of hosts during the summer drought (Domínguez Núñez et al., 2006; Morte et al., 2010). In a 2-year experiment, inoculation with *Tuber* spp. promoted the growth of *Quercus* spp. hosts in greenhouse (Wang et al., 2019). Our result probably implies that the inoculation plays a minor role only in the primary growth stages of plants grown in the favorable greenhouse environment, because 6 months are too short for the long lifespan of *Q. aliena*. Similar to *P. armandii* and *C. illinoinensis* inoculated with *Tuber* spp. and *T. sinoaestivum*, respectively (Zhang et al., 2020; Huang et al., 2021), the POD activity was higher in the seedlings inoculated with *T. melanosporum* than in the other treatments. In a previous study, POD activity was higher, and the incidence of pathogenic fungus *Microdochium tabacinum* was lower in soybeans inoculated with AMF fungi (Gao et al., 2018). Since the POD activity is one of the enzymatic defense mechanisms in plants (Ma et al., 2012), inoculation with *T. melanosporum* may possibly assist *Q. aliena* to confront infection.

In our study, the available phosphorus content of the ectomycorrhizosphere soils were higher in with truffle inoculation than without truffle inoculation, which was consistent with previous work (Li et al., 2017; Zhang et al., 2020). The higher concentration of phosphorus may be caused by the ectomycorrhizae (Dominguez et al., 2012) and the functional bacteria in ectomycorrhizosphere soils (Matthijs et al., 2007). Similar to previous studies (Kang et al., 2020; Siebyła and Hilszczañska, 2020), truffle inoculation did not affect the organic matter, total nitrogen, and available potassium contents, implying the *T. indicum* and *T. melanosporum* have limited influence on these properties of ectomycorrhizosphere soils. The nitrate-nitrogen contents were higher with than without truffle inoculation. Previous study showed that *T. melanosporum* mycelium prefer organic nitrogen than nitrate-nitrogen (Kamal, 2011), and this may be one cause of significantly more nitrate-nitrogen content in *T. melanosporum* ectomycorrhizosphere soils. Furthermore, PttNRT2.4A is a high affinity nitrate importer expressed in ectomycorrhizae, and the transcript levels PttNRT2.4A increased when ectomycorrhizae were exposed to low nitrate concentrations (10–100 μM) (Willmann et al., 2014). This may indicate that the nitrate-nitrogen content might be regulated by the level of nitrate transporter function gene in *Tuber* spp. ectomycorrhizae and the nitrate-nitrogen content in ectomycorrhizosphere soils. Furthermore, the higher total nitrogen content in *T. melanosporum* treatment than *T. indicum* treatment may due to the nitrate-nitrogen content.

Denitrifying bacteria are widely distributed in terrestrial ecosystems. Commonly, the *nirK*-type denitrifiers have been more abundant than *nirS*-type denitrifiers, e.g., in forest, paddy, and wetland soils and in the rhizosphere (Levy-Booth and Winder, 2010; Bannert et al., 2011; Hamonts et al., 2013). In agreement, the biodiversity and richness of *nirK*-type denitrifiers were greater than those of *nirS*-type denitrifiers in the rhizosphere of *Q. aliena* cultivated in greenhouse. In addition, the top abundant genera of *nirS-type* denitrifiers in rhizosphere soil of *Q. aliena* with *Tuber* spp. inoculation were the same as these in *C. illinoinensis* with *Tuber* spp. inoculation (Huang et al., 2021). Contrary to α-diversity, the β-diversity of *nirS*-type denitrifiers in ectomycorrhizosphere soils were different from rhizosphere soils, which may indicate that the *Tuber* spp. inoculation effect *nirS*-type denitrifier composition at species level. This needs further study with other DNA sequencing technology, such as metagenomics, to classify denitrifiers at species-level to reveal the character of denitrifiers composition in ectomycorrhizosphere soils.

The higher *nirK*-denitrifier richness in ectomycorrhizosphere soils was possibly associated with the higher nitrate-nitrogen content with truffle inoculation than without. Although the nitrate-nitrogen content was higher in *T. melanosporum* than *T. indicum* treatments, the *nirK*-denitrifier richness was lower in *T. melanosporum* than in *T. indicum* treatments. Among the *nirK*-denitrifiers detected in our study, *Bacillus*, *Nitrosomonas*, *Nitrosospira*, *Alcaligenes*, and *Pseudomonas* include many heterotrophic nitrifying–aerobic denitrifying bacterial strains, e.g., *Bacillus haynesii* (Huang et al., 2017), *Alcaligenes faecalis* (Joo et al., 2005; Zhao et al., 2012), *Pseudomonas* YY3 (Lang et al., 2019), *Nitrosomonas europaea* (Kozlowski et al., 2014), *N. europaea* (Chain et al., 2003), and *Nitrospira inopinata* (Van Kessel et al., 2015). Thus, the higher nitrate-nitrogen content in *T. melanosporum* ectomycorrhizosphere soils may have been partly due to nitrification activity. The *nirS*-type bacteria were more sensitive than *nirK*-type bacteria to anoxia, N limitation (Hamonts et al., 2013), and urine application (Anderson et al., 2014). In our study, the *nirS*-type bacteria correlated with NO_3_^–^-N and AP contents and pH, whereas the *nirK*-type bacteria correlated with the NO_3_^–^-N only, which may indicate that the *nirS*-type bacteria were more sensitive than the *nirK*-type bacteria to soil properties (Jones and Hallin, 2010; Azziz et al., 2017).

However, the *nirK*-denitrifiers were more sensitive to the inoculation. Baneras et al. (2012) concluded that the *nirK*-denitrifier community composition in rhizosphere soils varied with plant species, possibly due to the root exudates. *Tuber* spp. inoculation affected the compounds of *Q. mongolica* root exudates (Wang et al., 2021). In addition, ectomycorrhizae may secrete signal molecules belonging to diverse groups of organic compounds (Bouwmeester et al., 2007), and these metabolites present in the root-adhering soil were well-correlated with soil microbial denitrification activity and denitrifying bacterial community structure (Guyonnet et al., 2017; Hou et al., 2018). Compared to the uninoculated *Q. aliena*, the higher *nirK*-denitrifier diversity and richness in the rhizosphere of the inoculated seedlings might be due to difference in root exudates.

## Conclusion

We inoculated *T. melanosporum* and *T. indicum* on *Q. aliena* seedlings, determined the characteristics of ectomycorrhizae, seedlings and rhizosphere soil, and analyzed the rhizosphere *nirK*- and *nirS*-denitrifier communities. The higher POD activity in the seedlings inoculated with *T. melanosporum* than in the other treatments suggested that inoculation with *T. melanosporum* may possibly assist *Q. aliena* to confront infection. The AP contents were lower and nitrate-nitrogen nitrogen (NO_3_^–^-N) contents were higher with than without truffle inoculation, and *T. melanosporum* treatment differed more from the control than the *T. indicum* treatment. The diversity and richness of *nirK*-denitrifier communities were improved by two truffles inoculation, and the effect of *Tuber* spp. inoculation on the *nirS*-denitrifier communities was relatively small. The effect of inoculation on the soil properties and *nirK*- and *nirS*-denitrifier communities likely depends on the *Tuber* spp.-host plant combination.

## Data Availability Statement

The datasets presented in this study can be found in online repositories. The names of the repository/repositories and accession number(s) can be found below: https://www.ncbi.nlm.nih.gov/genbank/, PRJNA556350.

## Author Contributions

ZK, XL, and YG conceived and designed the experiments. ZK, BZ, and YL performed the experiments. ZK, XL, YL, XZ, PP, and YG wrote and revised the manuscript. All authors approved the final version of the manuscript.

## Conflict of Interest

The authors declare that the research was conducted in the absence of any commercial or financial relationships that could be construed as a potential conflict of interest.

## Publisher’s Note

All claims expressed in this article are solely those of the authors and do not necessarily represent those of their affiliated organizations, or those of the publisher, the editors and the reviewers. Any product that may be evaluated in this article, or claim that may be made by its manufacturer, is not guaranteed or endorsed by the publisher.

## Footnotes

1
http://sourceforge.net/projects/rdp-classifier/

2
http://fungene.cme.msu.edu/

3
https://www.r-project.org/

## Supplementary Material

Supplementary Figure 1 Rank abundance curves for **(A)** *nirK*- and **(B)** *nirS*-type denitrifying bacterial operational taxonomic unit (OTU) diversity in rhizosphere soil of not inoculation *Quercus aliena* (CK.ali) and *Q. aliena* inoculated with *Tuber melanosporum* (mel.ali) or *Tuber indicum* (ind.ali).

Supplementary Figure 2 Rarefaction curves of **(A)** *nirK*- and **(B)** *nirS*-OTUs in the rhizosphere of not inoculated *Q. aliena* (CK.ali) and *Q. aliena* inoculated with *Tuber melanosporum* (mel.ali) or *Tuber indicum* (ind.ali).

Supplementary Figure 3 The Shapiro-Wilk test for normality of **(A)** morphological indices and **(B)** physiological indices of *Quercus aliena* seedlings, **(C)** soil properties, and **(D)** richness and diversity of *nirK*- and *nirS*-denitrifiers.

Supplementary Figure 4 Differential abundance analysis of *nirK*- and *nirS*-OTUs in the rhizosphere of not inoculated *Q. aliena* (CK.ali) and *Q. aliena* inoculated with *Tuber melanosporum* (mel.ali) or *Tuber indicum* (ind.ali). **(A)** *nirK*-OTUs in CK vs. mel.ali, **(B)** *nirK*-OTUs in CK vs. ind.ali, **(C)** *nirK*-OTUs in ind.ali vs. mel.ali, **(D)** *nirS*-OTUs in CK vs. mel.ali, **(E)** *nirS*-OTUs in CK vs. ind.ali, and **(F)** *nirS*-OTUs in ind.ali vs. mel.ali. The x-axes show median log2 dispersion within treatments and the y-axes median log2 difference between treatments.
