# Supplementary figures and images for: Black Truffles Affect Quercus aliena Physiology and Root-Associated nirK- and nirS-Type Denitrifying Bacterial Communities in the Initial Stage of Inoculation
Source: Front Microbiol. 2022 Apr 28;13:792568. doi: 10.3389/fmicb.2022.792568 (PMC9096950; doi:10.3389/fmicb.2022.792568)

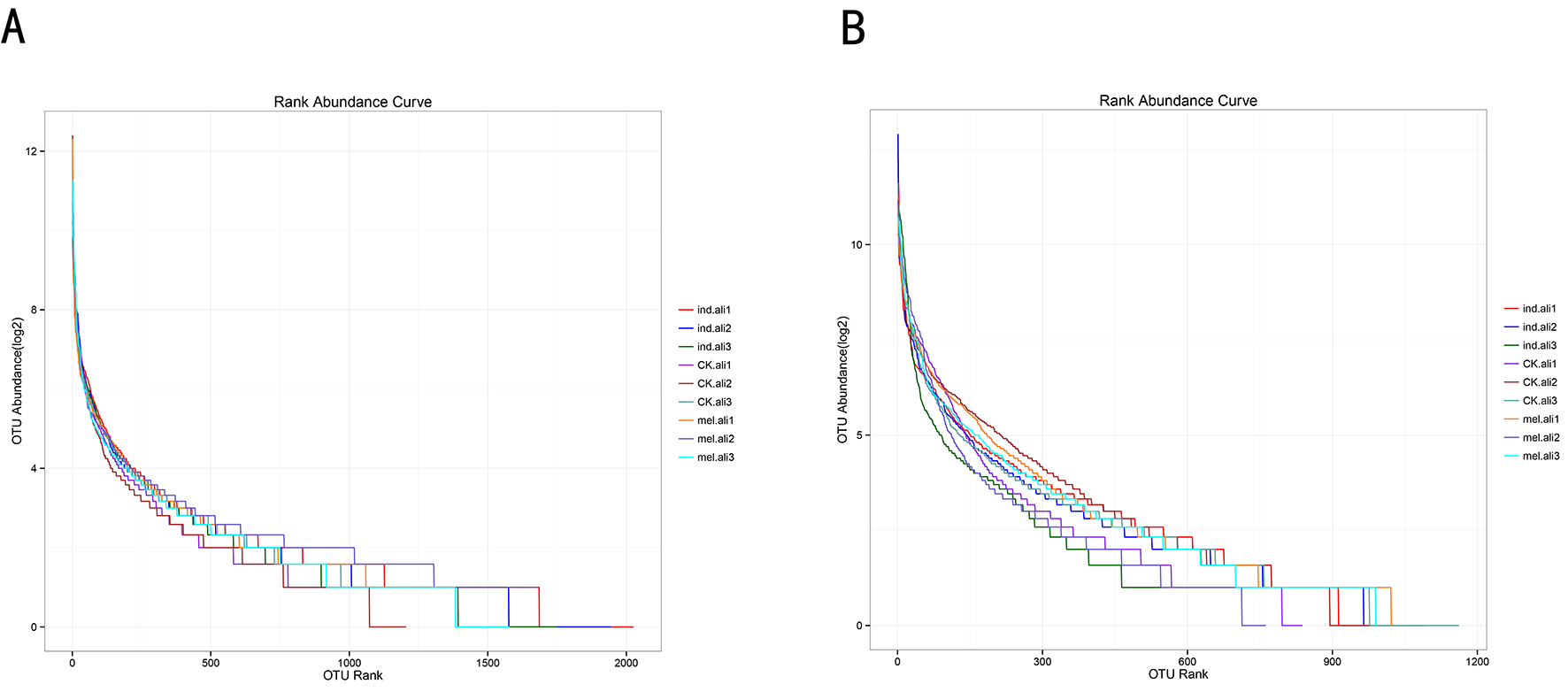

Supplement: Supplementary Figure 1 — Rank abundance curves for (A) nirK- and (B) nirS-type denitrifying bacterial operational taxonomic unit (OTU) diversity in rhizosphere soil of not inoculation Quercus aliena (CK.ali) and Q. aliena inoculated with Tuber melanosporum (mel.ali) or Tuber indicum (ind.ali). [file Image_1.tif]

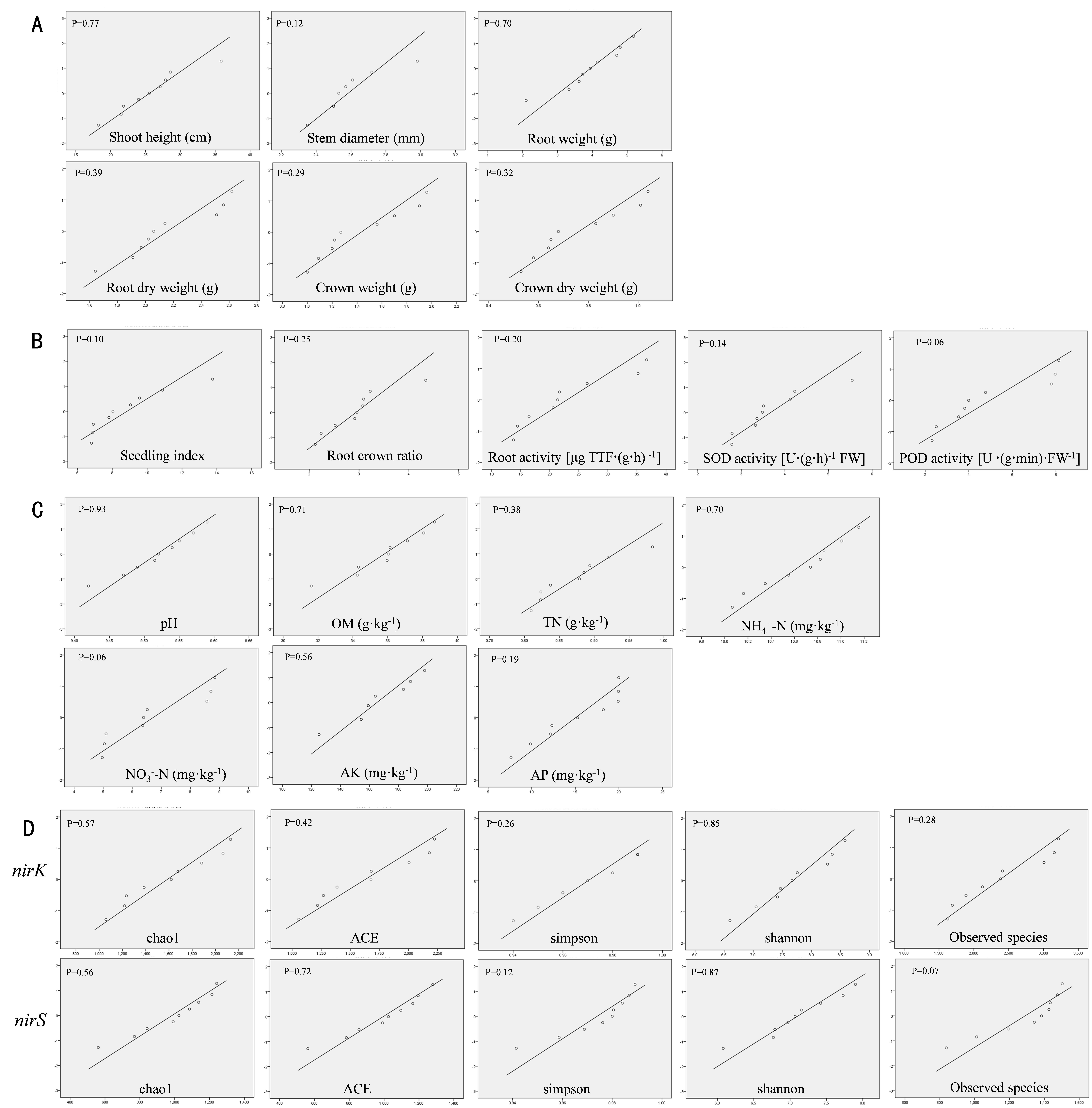

Supplement: Supplementary Figure 3 — The Shapiro-Wilk test for normality of (A) morphological indices and (B) physiological indices of Quercus aliena seedlings, (C) soil properties, and (D) richness and diversity of nirK- and nirS-denitrifiers. [file Image_3.tif]

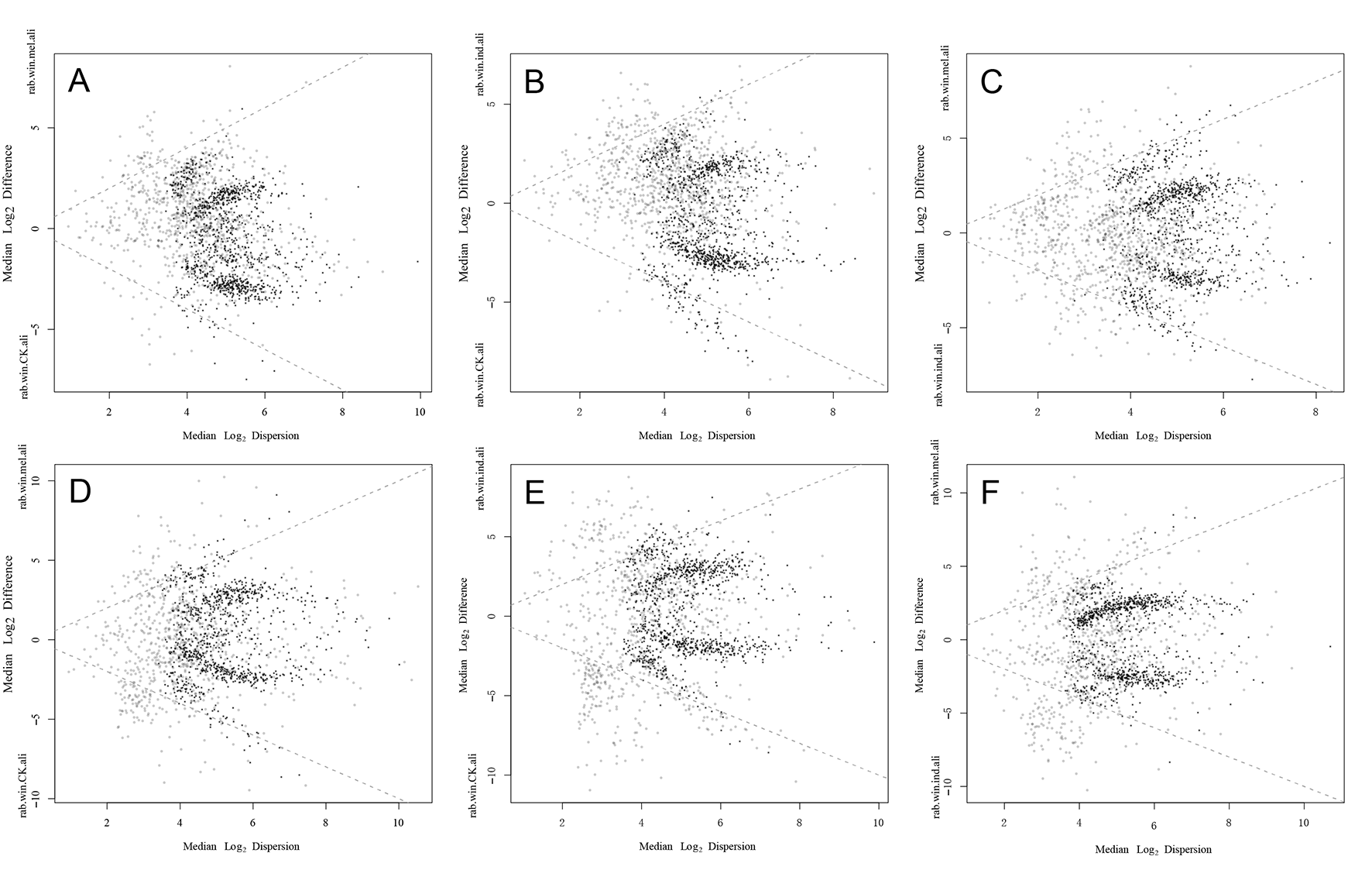

Supplement: Supplementary Figure 4 — Differential abundance analysis of nirK- and nirS-OTUs in the rhizosphere of not inoculated Q. aliena (CK.ali) and Q. aliena inoculated with Tuber melanosporum (mel.ali) or Tuber indicum (ind.ali). (A) nirK-OTUs in CK vs. mel.ali, (B) nirK-OTUs in CK vs. ind.ali, (C) nirK-OTUs in ind.ali vs. mel.ali, (D) nirS-OTUs in CK vs. mel.ali, (E) nirS-OTUs in CK vs. ind.ali, and (F) nirS-OTUs in ind.ali vs. mel.ali. The x-axes show median log2 dispersion within treatments and the y-axes median log2 difference between treatments. [file Image_4.tif]
