# Supplementary material for: Black Truffles Affect Quercus aliena Physiology and Root-Associated nirK- and nirS-Type Denitrifying Bacterial Communities in the Initial Stage of Inoculation
Source: Front Microbiol. 2022 Apr 28;13:792568. doi: 10.3389/fmicb.2022.792568 (PMC9096950; doi:10.3389/fmicb.2022.792568)
Supplement: Supplementary file 5 [file Table_1.docx]

**Supplementary Table 1** Morphological indices of *Q. aliena* seedlings.

| Treatments | Shoot height (cm) | Stem diameter (mm) | Root weight (g) | Root dry weight (g) | Crown weight (g) | Crown dry weight (g) |
| --- | --- | --- | --- | --- | --- | --- |
| CK.ali1 | 21.50 | 2.53 | 4.80 | 2.51 | 1.09 | 0.58 |
| CK.ali2 | 25.60 | 2.35 | 3.33 | 1.91 | 1.22 | 0.65 |
| CK.ali3 | 27.10 | 2.50 | 2.10 | 1.64 | 1.00 | 0.53 |
| mel.ali1 | 18.23 | 2.50 | 4.14 | 2.06 | 1.20 | 0.64 |
| mel.ali2 | 21.85 | 2.72 | 3.62 | 1.97 | 1.27 | 0.68 |
| mel.ali3 | 27.85 | 2.50 | 3.71 | 2.02 | 1.70 | 0.90 |
| ind.ali1 | 28.55 | 2.61 | 5.18 | 2.62 | 1.96 | 1.04 |
| ind.ali2 | 24.00 | 2.98 | 4.70 | 2.56 | 1.56 | 0.83 |
| ind.ali3 | 35.85 | 2.57 | 3.94 | 2.14 | 1.90 | 1.01 |

Note: Values are mean (*n=3*). CK.ali, rhizosphere soil of *Q. aliena* without *Tuber* partner; mel.ali, rhizosphere soil of *Q. aliena* with *T. melanosporum* partner; ind.ali, *Q. aliena* seedlings with *T. indicum* partner.
