# Supplementary material for: Black Truffles Affect Quercus aliena Physiology and Root-Associated nirK- and nirS-Type Denitrifying Bacterial Communities in the Initial Stage of Inoculation
Source: Front Microbiol. 2022 Apr 28;13:792568. doi: 10.3389/fmicb.2022.792568 (PMC9096950; doi:10.3389/fmicb.2022.792568)
Supplement: Supplementary file 6 [file Table_2.docx]

**Supplementary Table 2** The relative abundance of ten most abundant *nirK*- and *nirS*-type denitrifying bacterial genera in the rhizosphere soil of *Quercus aliena* with or without *Tuber* (*T. indicum* and *T.* *melanosporum*) partner.

| Samples | CK.ali | mel.ali | ind.ali |
| --- | --- | --- | --- |
| *nirK*-type denitrifying bacterial genera | *Achromobacter* (22.87%) | *Achromobacter* (26.13%) | *Bosea* (17.80%) |
|  | *Sinorhizobium* (22.47%) | *Sinorhizobium* (24%) | *Rhizobium* (15.33%) |
|  | *Paracoccus* (14.5%) | *Bosea* (10.8%) | *Achromobacter* (13%) |
|  | *Bosea* (9.43%) | *Devosia* (9.5%) | *Sinorhizobium* (12.6%) |
|  | *Devosia* (8.27%) | *Rhizobium* (8.5%) | *Devosia* (9.23%) |
|  | *Rhizobium* (3.57%) | *Ochrobactrum* (3.63%) | *Ochrobactrum* (5.43%) |
|  | *Ochrobactrum* (3.50%) | *Pseudomonas* (3.4%) | *Paracoccus* (5.3%) |
|  | *Rhodopseudomonas* (3.57%) | *Citrobacter* (2.87%) | *Citrobacter* (3.2%) |
|  | *Citrobacter* (1.77%) | *Alcaligenes* (2.47%) | *Pseudomonas* (2.7%) |
|  | *Alcaligenes* (1.53%) | *Paracoccus* (1.5%) | *Rhodopseudomonas* (2.47%) |
| *nirS*-type denitrifying bacterial genera | *Pseudomonas* (25.70%) | *Pseudomonas* (19.40%) | *Pseudogulbenkiania* (24.13%) |
|  | *Halomonas* (12.47%) | *Pseudogulbenkiania* (15.33%) | *Pseudomonas* (23.03%) |
|  | *Pseudogulbenkiania* (11.57%) | *Halomonas* (10.70%) | *Cupriavidus* (13.23%) |
|  | *Rhodanobacter* (10.10%) | *Sulfuritalea* (10.53%) | *Magnetospirillum* (5.57%) |
|  | *Sulfuritalea* (8.77%) | *Rhodanobacter* (7.80%) | *Rhodanobacter* (4.60%) |
|  | *Magnetospirillum* (6.90%) | *Cupriavidus* (6.67%) | *Halomonas* (3.63%) |
|  | *Cupriavidus* (6.00%) | *Herbaspirillum* (4.23%) | *Rubrivivax* (3.37%) |
|  | *Paracoccus* (2.60%) | *Azoarcus* (3.03%) | *Azoarcus* (2.67%) |
|  | *Rubrivivax* (2.3%) | *Magnetospirillum* (2.90%) | *Thauera* (2.60%) |
|  | *Azoarcus* (2.27%) | *Rubrivivax* (1.5%) | *Anaerolinea* (1.73%) |

Note: Values are mean (*n=3*). CK.ali, rhizosphere soil of *Q. aliena* without *Tuber* partner; mel.ali, rhizosphere soil of *Q. aliena* with *T. melanosporum* partner; ind.ali, *Q. aliena* seedlings with *T. indicum* partner.
